# Role of the cystathionine β-synthase / H_2_S pathway in the development of cellular metabolic dysfunction and pseudohypoxia in down syndrome

**DOI:** 10.1016/j.redox.2022.102416

**Published:** 2022-07-21

**Authors:** Theodora Panagaki, Laszlo Pecze, Elisa B. Randi, Anni I. Nieminen, Csaba Szabo

**Affiliations:** a Faculty of Science and Medicine, University of Fribourg, Fribourg, Switzerland; b Metabolomics Unit, Institute for Molecular Medicine Finland, University of Helsinki, Helsinki, Finland

**Keywords:** Down syndrome, Metabolism, Oxidative phosphorylation, Glycolysis, Hydrogen sulfide

## Abstract

**Background:**

Overexpression of the transsulfuration enzyme cystathionine-β-synthase (CBS), and overproduction of its product, hydrogen sulfide (H_2_S) are recognized as potential pathogenetic factors in Down syndrome (DS). The purpose of the study was to determine how the mitochondrial function and core metabolic pathways are affected by DS and how pharmacological inhibition of CBS affects these parameters.

**Methods:**

8 human control and 8 human DS fibroblast cell lines have been subjected to bioenergetic and fluxomic and proteomic analysis with and without treatment with a pharmacological inhibitor of CBS.

**Results:**

DS cells exhibited a significantly higher CBS expression than control cells, and produced more H_2_S. They also exhibited suppressed mitochondrial electron transport and oxygen consumption and suppressed Complex IV activity, impaired cell proliferation and increased ROS generation. Inhibition of H_2_S biosynthesis with aminooxyacetic acid reduced cellular H_2_S, improved cellular bioenergetics, attenuated ROS and improved proliferation. ^13^C glucose fluxomic analysis revealed that DS cells exhibit a suppression of the Krebs cycle activity with a compensatory increase in glycolysis. CBS inhibition restored the flux from glycolysis to the Krebs cycle and reactivated oxidative phosphorylation. Proteomic analysis revealed no CBS-dependent alterations in the expression level of the enzymes involved in glycolysis, oxidative phosphorylation and the pentose phosphate pathway. DS was associated with the dysregulation of several components of the autophagy network; CBS inhibition normalized several of these parameters.

**Conclusions:**

Increased H_2_S generation in DS promotes pseudohypoxia and contributes to cellular metabolic dysfunction by causing a shift from oxidative phosphorylation to glycolysis.

## 1 Background

Down syndrome (DS, caused by trisomy of chromosome 21) is the most common human genetic disorder, with a high prevalence of 1:700 births in developed countries. DS presents with cognitive impairment, craniofacial dysmorphism and co-morbidities such as cardiovascular defects and amyloid plaque pathology. The available therapeutic options for DS are limited and the clinical need for new therapeutic approaches is significant [1]. Although DS is associated with the dysregulation of thousands of genes and the dysregulation of numerous biochemical pathways, one of the underlying common themes is pseudohypoxia, i.e. a suppression of mitochondrial ATP generation in DS cells [2].

One of the genes that chromosome 21 codes for is the gene for cystathionine β-synthase (CBS, EC 4.2.1.22), a major enzyme in the transsulfuration pathway and one of the principal enzymes responsible for the synthesis of the gaseous transmitter hydrogen sulfide (H_2_S). H_2_S is now recognized as a significant mammalian biological regulator in health and disease [3,4]. As a result of the “gene dosage effect”, CBS is overexpressed in DS, and this has been long suspected to be a ‘master switch’ in the pathophysiology of DS [5,6]. However, its functional role in cellular and animal models of DS has only been directly confirmed in recent studies [7,8]. Our group has demonstrated that human DS fibroblasts exhibit a significant bioenergetic and mitochondrial defect, which is, to a large part due to the inhibitory effect of CBS-derived H_2_S on the activity of mitochondrial Complex IV [9]. Moreover, in rodent DS models that involve the triplication of CBS, the presence of CBS, and the generation of H_2_S has been causally linked to neurocognitive dysfunction [[7], [8]]. However, there are no studies which systematically investigate the cellular biochemical and bioenergetic alterations caused by CBS in DS. The current study investigates these mechanisms in a panel of human DS cells and healthy control cells *in vitro*.

## 2 Methods

*Materials.* Cell proliferation kit II (2,3-bis-(2-methoxy-4-nitro-5-sulphophenyl)–2H–tetrazolium–5–carboxanilide) (XTT; Ref ID: 11465015001), Cell Proliferation 5-bromo-2′-deoxyuridine (BrdU) colorimetric ELISA kit (Ref ID: 11647229001), and Cytotoxicity Detection Kit^PLUS^ (lactate dehydrogenase [LDH]; Ref ID: 4744934001) were purchased from Roche Diagnostics Ltd (Sigma-Aldrich Chemie GmbH, Buchs, Switzerland). The fluorescent H_2_S probes, 7-azido-4-methylcoumarin (AzMC) [10] and *(E)*-2-(3-(6-(2-hydroxyethylamino)naphthalen-2-yl)-3-oxoprop-1-enyl)-3,5-dimethoxybenzaldehyde (P3) [11], bovine serum albumin (BSA), d-glucose-^13^C_6_, and lactalbumin hydrolysate were purchased by Sigma-Aldrich Chemie GmbH. Agilent Seahorse XF Glycolytic Rate Assay (Ref ID: 103344–100) and Cell Mitostress Test (Ref ID: 103015–100) kits, Seahorse XF-24 cell culture microplates, and the corresponding Seahorse XF assay media and calibrant solution were purchased from Agilent Technologies (Bucher Biotech AG, Basel, Switzerland). The DCFDA/H_2_DCFDA ROS assay kit was purchased from Abcam PLC (Cambridge, UK). The human real-time PCR mitochondrial DNA damage analysis and mitochondrial DNA copy number kits were purchased from Detroit R&D (Holzel Diagnostika Handels GmbH, Köln, Germany). All other materials and reagents for cell culture and Western blotting were from Thermo Fisher Scientific (Basel, Switzerland), unless otherwise stated.

*Cell Culture.* Human dermal fibroblasts from healthy control subjects (CC) and dermal fibroblasts from individuals with Down syndrome (DSC) were obtained from LGC Standards (Wesel, Germany), the Coriell Institute for Medical Research (New Jersey, USA) and the Jérôme Lejeune Institute (Paris, France), as summarized in Table 1. Fibroblasts were cultured as previously described [9].

**Table 1:** **Description and origin of the human dermal fibroblasts used in the present study.** Control cells: CC; cells from individuals with Down syndrome: DSC; y: year; ∂: siblings; *: twins.

Reference ID	Group ID	Dot/Blot ID	Origin	Description	Gender	Age at sampling
CCD1064Sk	CC	C1	LGC Standards	Diploid	Male	<1y
3-FCYPR10000286*	CC	C2	Jérôme Lejeune Institute	Diploid	Male	5 y
GM05756	CC	C3	Coriell Institute	Diploid	Male	<1y
GM05659	CC	C4	Coriell Institute	Diploid	Male	1 y
Detroit 551	CC	C5	LGC Standards	Diploid	Female	<1y
1-FCYPR10000368^**∂**^	CC	C6	Jérôme Lejeune Institute	Diploid	Female	12 y
GM00041	CC	C7	Coriell Institute	Diploid	Female	<1y
GM00969	CC	C8	Coriell Institute	Diploid	Female	<1y
Detroit 532	DSC	D1	LGC Standards	Trisomy 21	Male	<1y
3-FCYPR10000285*	DSC	D2	Jérôme Lejeune Institute	Trisomy 21	Male	5 y
AG07096	DSC	D3	Coriell Institute	Trisomy 21	Male	<1y
AG05397	DSC	D4	Coriell Institute	Trisomy 21	Male	1 y
Detroit 539	DSC	D5	LGC Standards	Trisomy 21	Female	2 y
3-FCYPR10000369^**∂**^	DSC	D6	Jérôme Lejeune Institute	Trisomy 21	Female	9 y
GM02571	DSC	D7	Coriell Institute	Trisomy 21	Female	<1y
GM04616	DSC	D8	Coriell Institute	Trisomy 21	Female	<1y

*Cell Treatments.* Aminooxyacetic acid (AOAA) hemihydrochloride was purchased from Sigma-Aldrich Chemie GmbH and maintained in desiccated form till being used. AOAA was reconstituted in complete growth medium to a 10X working solution. From the latter, the added volume to the cell culture medium was adjusted to achieve the final concentration of 3 μM; control cells received the same volume of medium without AOAA. Treatment was initiated 2 h after cell seeding; measurements were made at 24 h.

*Cell Physiology Assays.* Cell Proliferation kit II (XTT), Cell Proliferation ELISA BrdU kit and Cytotoxicity Detection KitPLUS (LDH) were employed the quantification of fibroblast viability, proliferation, and necrosis, respectively. The assays were formatted in Corning® Costar® TC-Treated, flat-bottom, transparent 96-well microplates and performed as previously described [12,13].

*Quantification of H*_*2*_*S & ROS Levels in Live Cells.* All assays were formatted in Thermo Fisher Nunc® 96-well black, optical-bottom plates. Fluorescent signals were read with Infinite® 200 PRO microplate reader and imaged with the upright laser scanning confocal LEICA TCS SP5 BIO microscope. AzMC and P3 fluorophores were employed for the quantification of the intracellular H_2_S levels, as per our previously published methodology [12], while the cell-permeant reagent 2′,7′-dichlorofluorescein diacetate (DCFDA) was utilized for measuring measures hydroxyl, peroxyl, and other ROS species within the cell in accord to the manufacturer's protocol. Briefly, 24 h post the treatment with AOAA, fibroblasts were incubated with 20 μM DCFDA, 100 nM Invitrogen™ MitoTracker™ Deep Red FM and 1 μg/ml DAPI in assay buffer supplemented with 10% FBS for 1h at 37 °C in a humidified incubator with 5% CO_2_ and 95% air. The fluorescence signal of the DCFDA probe was detected at λ_excitation_ = 485 nm and λ_emission_ = 535 nm. A rise in the signal corresponds to increased oxidation of the intermediate deacetylated non-fluorescent product into the 2′, 7′—DCF catalyzed by ROS. MitoTracker™ signal was detected at λ_excitation_ = 580 nm and λ_emission_ = 644 nm. All fluorescent signals were double corrected to the total cell population of each condition (DAPI fluorescence; λ_excitation_ = 341 nm/λ_emission_ = 452 nm) and background (cell auto-fluorescence).

*Measurement of Mitochondrial Respiration.* Extracellular Flux (XF) analysis was employed for real-time quantification of oxygen consumption rate (OCR), proton efflux rate (PER) in intact cells; measurement of Complex IV activity was performed in permeabilized cells, using ascorbate and tetramethyl-*p*-phenylene diamine as Complex IV electron donors, according to our previously described methodology [9,12]. The assays were formatted in Seahorse XF24 cell-culture microplates, where diploid and 21-trisomic fibroblasts were seeded at a density of 2 × 10^4^ cells per well in total 200 μl for 2 h. Cells were subsequently treated with 0 or 3 μM AOAA for 24 h before the mitochondrial respiration being assessed.

*Mitochondrial DNA (mtDNA) Damage Assay.* Diploid and 21-trisomic fibroblasts were seeded at a density of 1 × 10^5^ cells/ml in total 5 ml in Corning® Costar® TC-Treated T25 flasks for 2 h. Cells were subsequently treated with 0 or 3 μM AOAA for 24 h. Followingly, cells were washed twice in pre-warmed 1X phosphate-buffered saline (PBS) formulated without Ca^2+^ and Mg^2+^, detached with TrypLE™ Express Enzyme, and pelleted by centrifugation at 200×*g* for 7 min. The cell pellet was used for DNA extraction with the DNAse Blood and Tissue Kit (Qiagen AG, Hombrechtikon, Switzerland) according to the manufacturer's recommendations. Mitochondrial DNA integrity was addressed with the commercially available human real-time PCR mitochondrial DNA damage analysis kit for the damaged amplicon 8.8 kb. Results were normalized to mitochondrial copy number and DNA integrity was expressed as a percentage of the mean value for the diploid, control fibroblasts. Both PCR assays were conducted according to the manufacturer's instructions.

Protein Extraction and Western Blotting. Diploid and 21-trisomic fibroblasts seeded at a density of 1 × 10^5^ cells/ml in total 10 ml in Corning® Costar® TC-Treated T75 flasks for 2 h. Cells were subsequently treated with 0 or 3 μM AOAA for 24 h. Followingly, cells were washed twice with ice-cold 1X PBS formulated without Ca^2+^ and Mg^2+^ and harvested in 1X PathScan® Sandwich ELISA cell lysis buffer (Cell Signaling Technology, BioConcept AG, Allschwill, Switzerland) supplemented with Halt™ protease/phosphatase inhibitor cocktail (1X). After two freeze/thaw cycles, the whole-cell lysate was collected and sonicated for 1 min (5 s ON/5 s OFF-6 cycles). Whole-cell proteins were extracted, quantified, handled and processed for Western blotting as previously published [9,12].

^*13*^*C Metabolic Flux Analysis.* CCs and DSCs were seeded at a density of 1 × 10^5^ cells/ml in total 30 ml of SILAC Advanced DMEM/F-12 Flex specially supplemented with 3 g/l stable isotopic labeled d-Glucose-^13^C_6_ (in addition to the other media supplements described above) in Corning® Costar® TC-Treated T175 flasks for 2 h. Cells were subsequently treated with 0 or 3 μM AOAA for 24 h. Followingly, cells were washed twice in ice-cold 1X PBS formulated without Ca^2+^ and Mg^2+^, detached by mechanical formulated without Ca^2+^ and Mg^2+^, harvested by cell scraping, and pelleted by centrifugation at 4000 rpm for 5 min at 4 °C. Cell pellet was snap-frozen and stored at −80 °C till processed for analysis of ^13^C-labeling of cellular metabolites as described [[14], [15], [16]]. Metabolites were extracted from cell using 400 μl of cold extraction solvent (acetonitrile:methanol:MQ, 40:40:20). Subsequently, samples were vortexed for 2 min and sonicated 1 min (settings: sweep mode, frequency 37, power 60, no heating) followed by centrifugation at 14,000 rpm at 4 °C for 5 min. Supernatants were dried with nitrogen gas and finally resuspended to 40 μl of extraction solvent, vortexed for 2 min and 2 μl of sample was injected to Thermo Vanquish UHPLC coupled with Q-Exactive Orbitrap mass spectrometer equipped with a heated electrospray ionization (H-ESI) source probe (Thermo Fischer Scientific). A SeQuant ZIC-pHILIC (2.1 × 100 mm, 5-μm particle) column (Merck) was used for chromatographic separation. Gradient elution was carried out with a flow rate of 0.100 ml/min with using 20 mM ammonium hydrogen carbonate, adjusted to pH 9.4 with ammonium solution (25%) as Mobile Phase A and acetonitrile as Mobile Phase B. The gradient elution was initiated from 20% Mobile Phase A and 80% of Mobile Phase B and maintain till 2 min, after that 20% Mobile Phase A gradually increased up to 80% till 17 min, then 80%–20% Mobile Phase A decreased in 17.1 min and maintained up to 24 min. The column oven and auto-sampler temperatures were set to 40 ± 3 °C and 5 ± 3 °C, respectively.

MS equipped with a heated electrospray ionization (HESI) source was used with polarity switching and following setting: resolution of 35,000, the spray voltages: 4250 V for positive and 3250 V for negative mode, the sheath gas: 25 arbitrary units (AU), and the auxiliary gas: 15 AU, sweep gas flow 0, Capillary temperature: 275 °C, S-lens RF level: 50.0. Instrument control was operated with the Xcalibur 4.1.31.9 software (Thermo Fischer Scientific). The peak integration was done with the TraceFinder 4.1 software (Thermo Fischer Scientific) using confirmed retention times for metabolites (m+0) standardized with library kit MSMLS-1EA (Merck). ^13^C isotopologolues were analyzed with TraceFinder 4.1 with responding *m/z* (m+1, m+2 etc.) in compound library. Data quality was monitored throughout the run using pooled QC sample prepared by pooling 5 μl from each suspended samples and interspersed throughout the run as every 10^th^ sample. The metabolite data was checked for peak quality, %RSV and carryover. Each metabolite peak area was normalized to the cell lysate DNA content. Relative abundance values were log 2 normalized. Four groups (n = 8 CC, n = 8 CC + AOAA, n = 8 DSC and n = 8 DSC + AOAA) were included in the analysis.

*Proteomics Analysis.* Proteomics data were then generated from a total of 32 samples using LC/MS labels with tandem mass tags at Charles River Laboratories (CRL) Little Chesterford, UK. The initial data matrix consisted of protein intensities for 7485 proteins in a total of 40 samples (8 pool samples + 32 human donor samples). Initial processing revealed that 1947 proteins had no intensity readings in at least 2 out of 8 replicates in any one of the four sample groups. These proteins were therefore excluded from analysis, leaving intensity values for 5538 proteins remaining for further analysis. Of the remaining proteins that still contained missing values, imputation was performed for these using the LOD2 approach. Automatic outlier tests using Euclidean distance, Kolmogorov-Smirnov, correlation, and Hoeffding's D were performed on this batch corrected dataset. No samples were identified as outliers; therefore all samples were retained for inclusion in downstream analysis. Significantly differentially expressed proteins were determined using linear modelling, as implemented in the Bioconductor package limma. REACTOME pathway and Gene Ontology (GO) analyses were performed using genes encoding the differentially expressed proteins identified (at the false discovery rate-adjusted p ≤ 0.1 threshold). Protein values were log2 normalized. Four groups (n = 8 CC, n = 8 CC + AOAA, n = 8 DSC and n = 8 DSC + AOAA) were included in the analysis.

*Data analysis and statistical procedures.* For the design of the current study group sizes were designed to be equal. Blinding was undertaken in the analysis of the data: cells were cultured and treated with pharmacological inhibitors by one investigator who has also performed randomization of the samples; functional (e.g. bioenergetic, fluxomic) analysis was conducted by other investigator(s) not aware of the groups and treatments. Results were expressed as mean ± standard error (SEM) of 8 independent pairs of diploid and 21-trisomic fibroblasts, as listed in Table 1. Exact group sizes (n) are provided in all figures and tables with group size referring to biological samples (i.e. each data point is a separate, different cell type from a different donor) – i.e. not a technical replicate. When justified, data pairs (i.e. the same cell with/without pharmacological inhibitor) are highlighted connected with a dotted line. Sex was considered as an experimental variable; the sex of the donors used in the study is shown in Table 1; no significant sex-dependent differences were observed in the study, consistent with the body of prior data showing that the biochemical alterations in DS are predominantly independent of sex. The fluxomic and proteomic sets were log2 transformed and quantile normalized; the main rationale for this transformation being heteroskedasticity: the variance of expression measurements on many platforms (arrays, etc.) depends on the expression level. By log-transforming, this dependence is reduced the data are more suitable for statistical testing. Two-way ANOVA analysis, followed by *post-hoc* Bonferroni's multiple-comparison *t*-test was used to identify differences among groups of treated and untreated conditions. Post-hoc tests were conducted only if F was significant and there was no variance in homogeneity. Differences among means were considered significant when p < 0.05. Statistical calculations were performed using GraphPad Prism 8 (GraphPad Software Inc., San Diego, USA). No data were excluded from any analysis.

## 3 Results and discussion

**Fig. 1:**
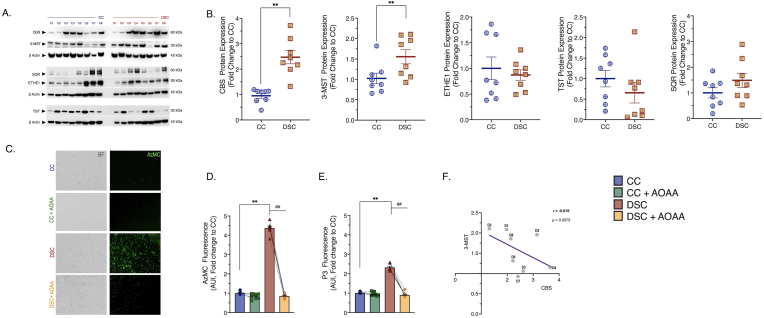
**DS is associated with CBS and 3-MST overexpression in human dermal fibroblasts. (A)** & **(B)**: Protein expression of the H_2_S-producing enzymes – CBS and 3-MST, and of the H_2_S-catabolizing enzymes ETHE1, SQR, and TST, as quantified by immunoblotting. β-actin served as loading control for densitometry. **(C):** Representative images of the fluorescent signal of the H_2_S-specific probe, AzMC along with **(D)** its quantification under baseline (untreated) conditions and following 24-h treatment with 3 μM AOAA. **(E)**: Quantification of cellular H_2_S levels using a second H_2_S probe, P3. **(F)**: Correlation between CBS and 3-MST enzyme expression patterns in DS. Each line and bar graph represents the mean ± SEM of n = 8 human euploid control fibroblasts and n = 8 DS fibroblasts. C1–C8 and D1-D8 corresponds to the specific donors listed in Table 1. Dotted connecting lines in the bar graphs indicate the same cell from a specific donor with/without AOAA treatment. **p ≤ 0.01 DSC indicates significant differences between untreated vs. CC untreated; ^##^p ≤ 0.01 indicates significant differences between DSC + AOAA vs. DSC untreated. The immunoblot for 3-MST along with the corresponding loading control has been previously published [13] and is reused with permission and according to the Open Access Policy of the journal.

We have assembled a panel of 8 human control and 8 human DS fibroblast cell lines (Table 1) for the current investigation. First, we have assessed the potential overactivation of the CBS/H_2_S pathway in DS, based on the ‘gene dosage’ effect (since CBS is encoded on human Chromosome 21) [7]. As expected, there was a marked upregulation of CBS in the DS cells, although there was significant variability in the degree of this upregulation (Fig. 1A, Fig. 1B). As previously reported [13], although not encoded on Chromosome 21, the expression of a second, major mammalian H_2_S- and polysulfide-producing enzyme, 3-mercaptopyruvate sulfurtransferase (3-MST) was also higher in the DS fibroblasts than in the control cells (Fig. 1A and B). The H_2_S-metabolizing enzymes SQR, ETHE-1 and TST did not show any difference between control and DS cells (Fig. 1A and B). There was a marked and uniform increase in H_2_S generation in DS cells as demonstrated by live cell imaging by two structurally different H_2_S fluoroprobes (Fig. 1C, D, Fig. 1E). There appeared to be a weak inverse correlation between the degree of CBS and 3-MST expression in DS cells (Fig. 1F).

**Fig. 2:**
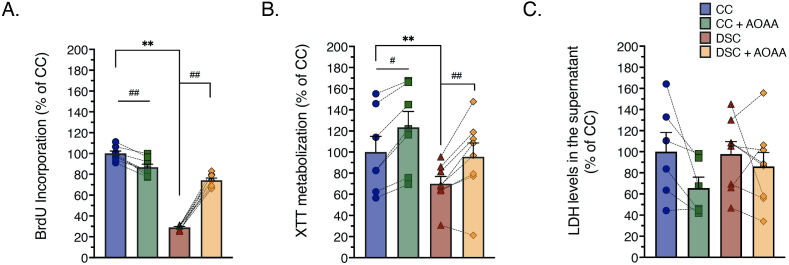
**Pharmacological inhibition of CBS with AOAA restores the suppressed cell proliferation and viability of DS fibroblasts, without adversely affecting cell viability.** Quantification of **(A)**: BrdU incorporation, a marker of cell proliferation, **(B)**: XTT metabolization, a marker of mitochondrial function and overall cell viability and **(C)**: LDH levels in the supernatant, a marker of cell necrosis and the breakdown of the cell membrane permeability were assessed. Each line and bar graph represents the mean ± SEM of n = 8 human euploid control fibroblasts and n = 8 DS fibroblasts, as summarized in Table 1. Dotted connecting lines indicate the same cell from a specific donor with/without AOAA treatment (3 μM, 24 h). **p ≤ 0.01 indicates significant differences between DSC untreated vs. CC untreated; ^#^p ≤ 0.05 and ^##^p ≤ 0.01 indicate significant differences between CC + AOAA vs. CC untreated or DSC + AOAA vs. DSC untreated.

In line with prior findings [9,13] DS cells proliferated slower than control fibroblasts (Fig. 2A), and exhibited a decreased ability to convert the dye XTT to formazan (a mitochondrion-dependent function) (Fig. 2B). However, no significant cell necrosis (breakdown of the cell membrane, and LDH release into the supernatant) was noted in the DS fibroblasts (Fig. 2C), indicating that these cells, even if their mitochondrial function is impaired, are able to produce sufficient amounts of ATP to maintain membrane integrity [14]. When treated with aminooxyacetic acid (AOAA, 3 μM, 24 h), a commonly used pharmacological inhibitor of CBS [3], DS cell proliferation was stimulated; an effect that was uniform in its direction and magnitude across the 8 DS cells studied. In contrast, in control cells AOAA did not increase cell proliferation, but, instead, produced a slight decrease (Fig. 2A) while it slightly increased XTT conversion (Fig. 2B). Taken together, in control cells H_2_S does not appear to play a large role in modulating mitochondrial function and proliferation, while in DS cells, the elevated H_2_S levels exerts inhibitory effects on mitochondrial function and cell proliferation. Indeed, H_2_S has a well-documented ability to inhibit mitochondrial Complex IV, leading to the suppression of cellular bioenergetics and cell viability; an action which has been well characterized by earlier environmental toxicological and biochemical studies [3,4,17,18].

**Fig. 3:**
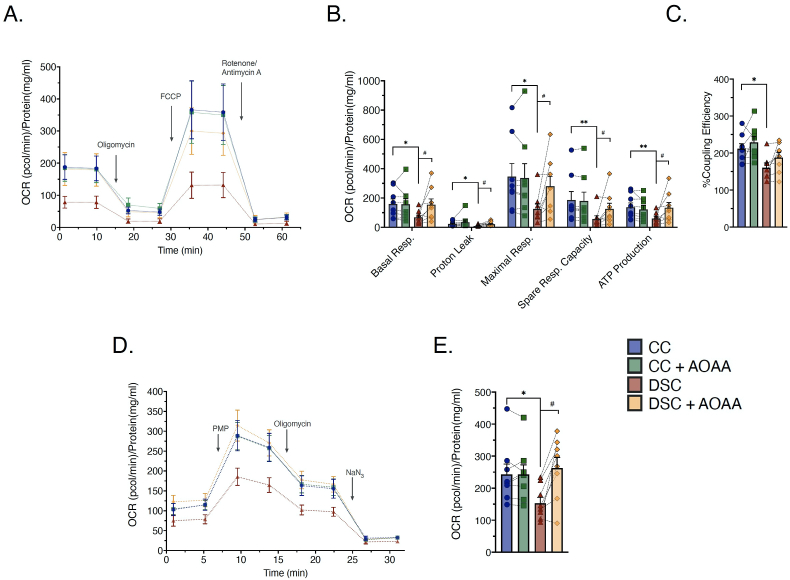
**Mitochondrial O**_**2**_**consumption and specific mitochondrial electron transport Complex IV activity is suppressed in DS fibroblasts; pharmacological inhibition of CBS with AOAA improves the bioenergetic phenotype of DS cells without significantly affecting the bioenergetics of control cells. (A)**: Rates of oxygen consumption rate (OCR) recorded before and after sequential addition of oligomycin (1 μM), FCCP (2 μM) and rotenone/antimycin A (0.5 μM) to the cells using the Agilent Seahorse XFe24 Analyzer. **(B)**: Calculated mean values of basal respiration, proton leak, maximal respiration, spare respiratory capacity, ATP-linked respiration. **(C)**: Calculated mitochondrial coupling efficiency. **(D)** & **(E)** Specific activity of mitochondrial Complex IV in permeabilized cells. (PMP depicts the administration of the Agilent Seahorse XF Plasma Membrane Permeabilizer, a proprietary reagent that permeabilizes intact cells in culture.) Each line and bar graph represents the mean ± SEM of n = 8 human euploid control fibroblasts and n = 8 DS fibroblasts, as summarized in Table 1. Dotted connecting lines in the bar graphs indicate the same cell from a specific donor with/without AOAA treatment (3 μM, 24 h). *p ≤ 0.05, **p ≤ 0.01 indicates significant differences between DSC untreated vs. CC untreated; #p ≤ 0.05 indicates significant differences between DSC + AOAA vs. DSC untreated.

Extracellular Flux Analysis studies showed that DS cells exhibited a marked decrease in mitochondrial electron transport, ATP generation and coupling efficiency compared to control cells (Fig. 3A,B,C). In line with the prior analysis [9], this effect is due to the inhibition of mitochondrial Complex IV activity in DS cells (Fig. 3D and E). In control cells AOAA did not affect the bioenergetic parameters, but in DS cells it increased various bioenergetic parameters related to mitochondrial oxidative phosphorylation (Fig. 3). These findings confirm and extend our earlier findings, obtained in comparison with a single pair of control and DS fibroblasts (Detroit 551 vs. 539) [9], and suggest that a reversible inhibition of Complex IV by increased H_2_S contributes to the mitochondrial and overall bioenergetic deficit. Inhibition of aerobic O_2_ consumption and coupling efficiency in DS fibroblasts was also reported recently in a study [19] conducted by Anderson and colleagues at the University of Colorado, by comparing 14 healthy and 14 DS fibroblasts (using DS populations that are different from those used in the current study). The effect of AOAA in reversing the bioenergetic suppression in DS cells was somewhat variable across all 8 DS cell lines investigated (Fig. 3), indicative that the CBS/H_2_S component of metabolic suppression shows individual variation across DS, perhaps as a function of different degree of overexpression of H_2_S-producing enzymes, and/or individually different counterregulatory and compensatory mechanisms.

**Fig. 4:**
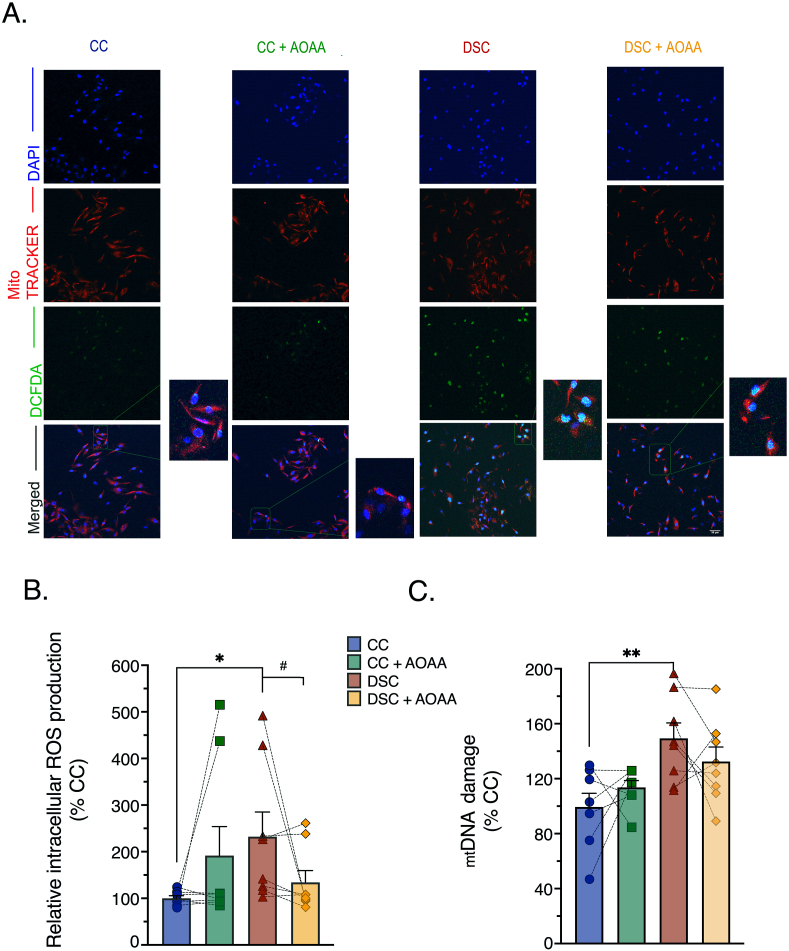
**Pharmacological inhibition of CBS with AOAA attenuates the DS-associated overproduction of reactive oxygen species (ROS) and ameliorate mitochondrial DNA damage (mtDNA). (A)**: Representative pictures of control cells (CC) and dermal fibroblasts from individuals with Down syndrome (DSC) labeled with the redox-sensitive probe 2′,7’ –dichlorofluorescein diacetate (DCFDA) under basal (untreated) conditions and in the presence of AOAA treatment. **(B)**: Quantitative analysis of the DCFDA fluorescent signal. **(C)**: Assessment of mitochondrial DNA damage, expressed as a fold change to CC after baseline normalization to the corresponding mitochondrial copy number. Each bar graph represents the mean ± SEM of n = 8 human euploid control fibroblasts and n = 8 DS fibroblasts, as summarized in Table 1. Dotted connecting lines in the bar graphs indicate the same cell from a specific donor with/without AOAA treatment (3 μM, 24 h). *p ≤ 0.05, **p ≤ 0.01 indicate significant differences between DSC untreated vs. CC untreated; ^#^p ≤ 0.05 indicates significant differences between DSC + AOAA vs. DSC untreated.

When electron flux over the mitochondrial electron transport chain is blocked, electrons often “leak off” from these complexes to react with oxygen to produce various oxidants and free radicals [20]. Indeed, prior studies have already demonstrated an increase in ROS generation in DS cells [[21], [22], [23], [24]]. In line with these observations, DS cells exhibited an intense 2′,7′-dichlorofluorescein (DCF) signal brightly colocalized with MitoTracker™ staining, signaling for an increased mitochondrial ROS generation (Fig. 4A and B). There was more mitochondrial DNA damage in DS than in control cells (Fig. 4C), in accordance with prior studies [19,25,26] and in line with the sensitivity of the mitochondrial DNA to ROS-mediated oxidative damage [[27], [28], [29]]. In line with its beneficial effects on mitochondrial electron transport, AOAA normalized ROS generation (Fig. 4A and B) and improved mitochondrial DNA integrity (Fig. 4C) in DS cells.

**Fig. 5:**
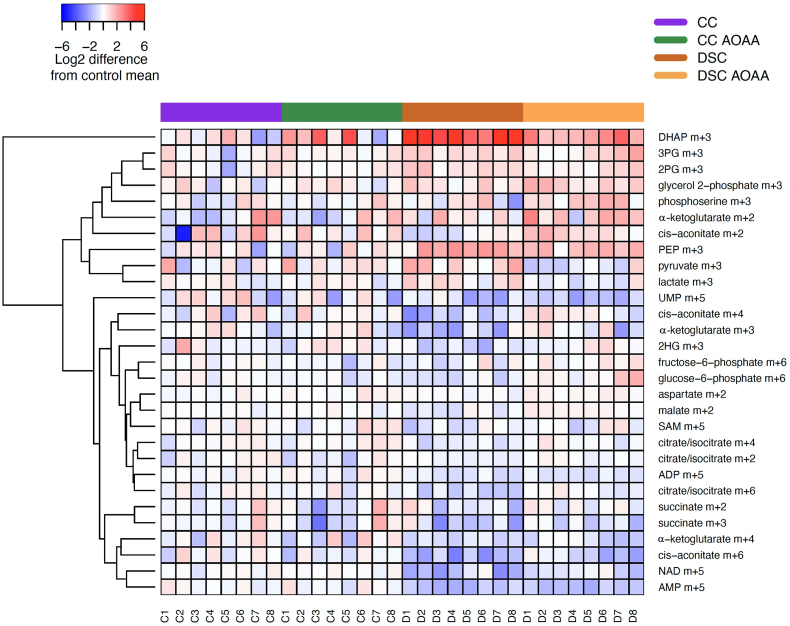
**DS cells show a markedly altered fluxomic profile compared to healthy control cells; effect of AOAA.** The heat map shows the fluxomic alterations in various metabolites of n = 8 human euploid control fibroblasts and n = 8 DS fibroblasts, as summarized in Table 1 with/without AOAA treatment (3 μM).

**Fig. 6:**
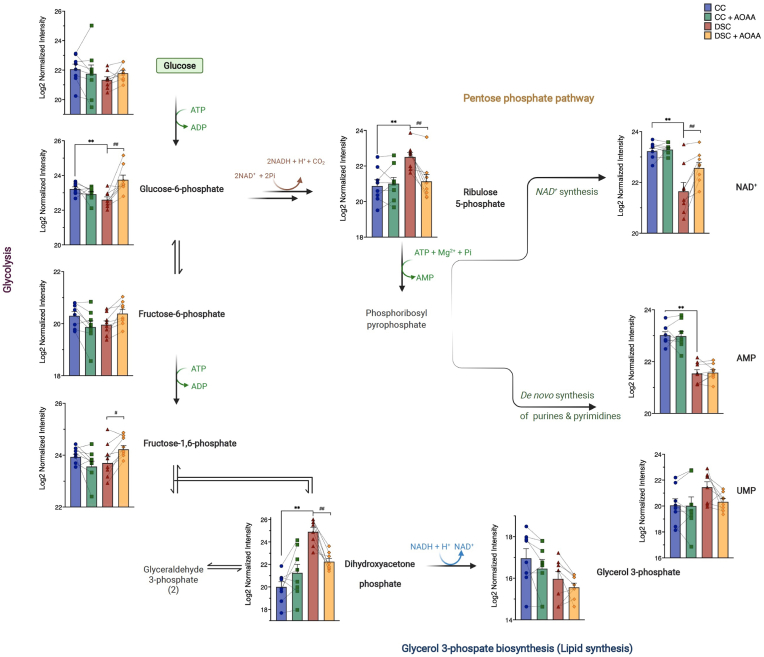
**DS cells exhibit a markedly altered glycolytic and pentose phosphate pathway (PPP) fluxomic profile compared to healthy control cells; effect of AOAA.** Isotopologues present in the initial steps of glycolysis and the pentose phosphate pathway, as well as in *de novo* synthesis of pyrimidines and purines and in the initial steps of lipid biosynthesis are shown in n = 8 human euploid control fibroblasts and n = 8 DS fibroblasts, as summarized in Table 1, with/without AOAA treatment (3 μM). **p ≤ 0.01 indicates significant differences between DSC untreated vs. CC untreated; ^##^p ≤ 0.01 indicates significant differences between DSC + AOAA vs. DSC untreated.

**Fig. 7:**
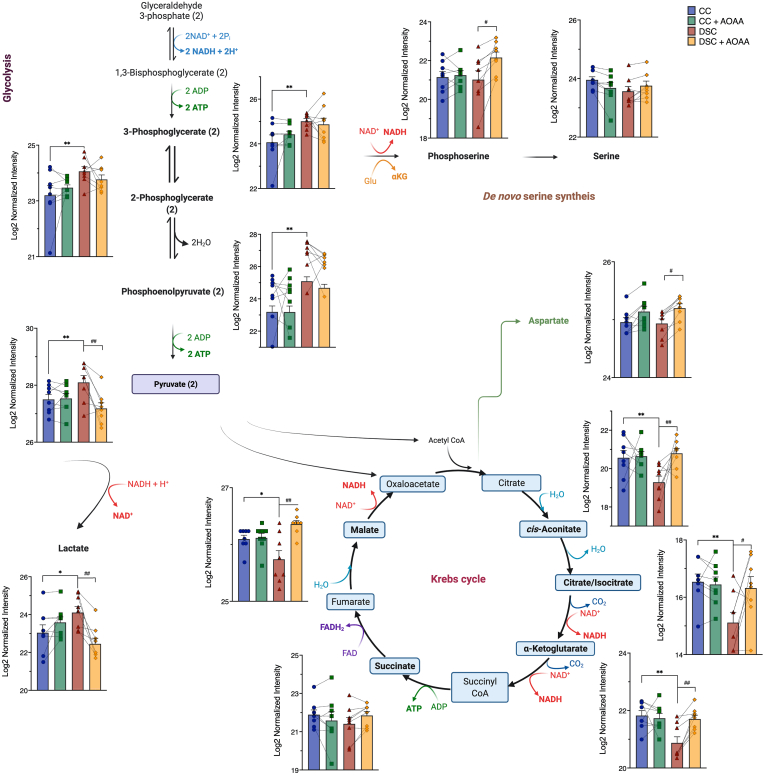
**DS cells exhibit a markedly enhanced glycolytic and impaired Krebs cycle fluxomic profile compared to healthy control cells; effect of AOAA.** Isotopologues present in the distal steps of glycolysis and the Krebs cycle, as well as steps of serine biosynthesis are shown in n = 8 human euploid control fibroblasts and n = 8 DS fibroblasts, as summarized in Table 1, with/without AOAA treatment (3 μM). **p ≤ 0.01 indicates significant differences between DSC untreated vs. CC untreated; ^#^p ≤ 0.05, ^##^p ≤ 0.01 indicates significant differences between DSC + AOAA vs. DSC untreated.

In order to obtain further insight into the metabolic alterations associated with DS, we next employed fluxomics analysis. Addition of ^13^C-labeled glucose to cell culture medium leads to time-dependent incorporation of ^13^C into various metabolic intermediates and allows the detection of metabolic fluxes into various core bioenergetic pathways – including glycolysis, Krebs cycle and the pentose phosphate shunt [30]. We have observed marked differences between control and DS cells that are consistent with a suppression of mitochondrial electron transport demonstrated above. Importantly, DS cells, in comparison to control cells, exhibited a marked suppression of ^13^C fluxes into the Krebs cycle (Fig. 5, Fig. 6, Fig. 7). At the same time, a significant (likely compensatory) increase in carbon fluxes was observed into glycolysis metabolites (Fig. 5, Fig. 6, Fig. 7). The increased glycolytic activity of DS cells was evidenced by increased levels of the isotopologues of glycerol-3-phosphate (G3P), dihydroxyacetone phosphate (DHAP), 3-phosphoglycerate (3-PG), 2-phosphoglycerate (2-PG), phosphoenolpyruvate (PEP), and pyruvate.

In healthy cells with intact mitochondrial electron transport and in the presence of sufficient O_2_ supply, pyruvate enters the Krebs cycle. However, when the Krebs cycle is inhibited, it is reduced to lactate. The increased levels of lactate isotopologues in DS cells (Fig. 5) indicates that DS cells are in a pseudohypoxic state, a finding already predicted by a prior meta-analysis [3]. The increased flux of glucose-derived ^13^C carbons into lactate shown in the current study is consistent with biochemical and clinical observations demonstrating an increased lactate production and a decreased exercise tolerance in DS individuals [1,2].

In contrast to the enhanced glycolytic fluxes, many Krebs cycle metabolites (citrate, *cis*-aconitate, alpha-ketoglutarate, malate) exhibited reduced fluxes in DS cells (Fig. 7). These data indicate that in DS cells the mitochondrial electron transport chain's activity is suppressed, and, consequently, the generation of electron donors to feed this activity (the fundamental function of the Krebs cycle) is consequently also decreased. Although glycolysis is a less efficient way to generate ATP than oxidative phosphorylation (2 molecules vs. 36 molecules of ATP per glucose consumed), the speed of glycolysis (i.e. the number of glucose molecules utilized over a given time) is substantially faster than that of oxidative phosphorylation [31,32]. We believe that this is the reason why cellular energy charge is not more drastically depleted in DS cells, and the likely reason while the DS cells, in fact, are able to maintain fundamental baseline cellular functions, such as the maintenance of membrane potential and cell division. We hypothesize that the above shift from oxidative phosphorylation to glycolysis may explain why DS individuals exhibit reduced exercise tolerance [1,2]: for a cell that is “fueled” by a large part by glycolysis, it is difficult to mobilize additional ATP generation to meet sudden increases in cellular metabolic demand.

Interestingly, DS was associated with a significant increase in ^13^C fluxes into ribose/ribulose-5-phosphate (Ri/Ru5P), indicative of an upregulation of the pentose-phosphate pathway (PPP) in DS (Fig. 6). Via this pathway, it is possible that DS cells may generate NADH, since (as shown above), the activity of the Krebs cycle is impaired. Indeed, prior observations in cancer cells have already suggested that CBS activation or forced overexpression can induce a metabolic shift into the PPP [33,34]. Moreover, the fluxomic analysis revealed a DS-associated increase in the ^13^C fluxes into glycerol-3-phosphate (G3P) from DHAP (Fig. 6).

Importantly, AOAA restored many of the above-listed fluxomic shifts in DS cells; it restored the DS-associated suppression of the ^13^C fluxes into various Krebs cycle intermediates (citrate, *cis*-aconitate, α-ketoglutarate, succinate, malate and aspartate) while it reduced fluxes into the terminal glycolytic intermediates (phosphoenolpyruvate [PEP] and pyruvate). These actions culminated in the normalization of the DS-related increases in ^13^C flux to lactate, suggesting that inhibition of CBS can restore the activity of the Krebs cycle in DS cells, and, consequently, it can attenuate the DS-associated compensatory stimulation of glycolysis (Fig. 5, Fig. 6, Fig. 7). According to our working hypothesis DS leads to an inhibition of the Krebs cycle, because the cell is unable to utilize the Krebs-cycle-derived electron donors due to the inhibition of mitochondrial electron transport by excess H_2_S. Therefore, pyruvate does not enter the Krebs cycle and essentially shuts it off (thus, there is a decrease in all metabolites of the cycle). CBS inhibition “lifts” the H_2_S-mediated inhibition of Complex IV, restores mitochondrial electron transport; consequently, the Krebs-cycle-derived electron donors are, once again, utilized normally. Thus, the activity of the Krebs cycle, and the entry of pyruvate into the Krebs cycle are restored. Interestingly, in a previous study [35], in MIN6 cells subjected to the endoplasmic reticulum stress inducer thapsigargin, inhibition of cellular H_2_S biosynthesis also attenuated the stress-induced increases in ^13^C fluxes into lactate, while – again, similar to our findings – inhibition of cellular H_2_S biosynthesis also restored the suppressed ^13^C fluxes through the Krebs cycle.

AOAA treatment of DS cells also normalized the DS-associated upregulation of ^13^C flux into the proximal (oxidative) phase of PPP and normalized the DS-associated increase in the ^13^C fluxes into glycerol-3-phosphate (G3P) from DHAP (Fig. 6). Treatment of DS cells with AOAA produced an additional fluxomic response with potential biological significance: a partial restoration of the DS-associated suppression of *de novo* NAD^+^ synthesis (Fig. 6). The effect of AOAA was DS-selective; the inhibitor did not affect ^13^C fluxes into NAD^+^ synthesis in healthy control cells. While the biochemical mechanism underlying this effect remains to be further explored, we hypothesize that via this effect CBS inhibition may improve the activity of various NAD^+^-dependent enzymes in DS.

AOAA in DS cells increased ^13^C flux into glutamate, but a similar AOAA-induced increase was also observed in normal control cells (Fig. 7). Glutamate has important roles both as a metabolic factor, a building block of protein synthesis, as well as an excitatory amino acid, and the depletion of which has been implicated in the pathogenesis of DS [36].

**Fig. 8:**
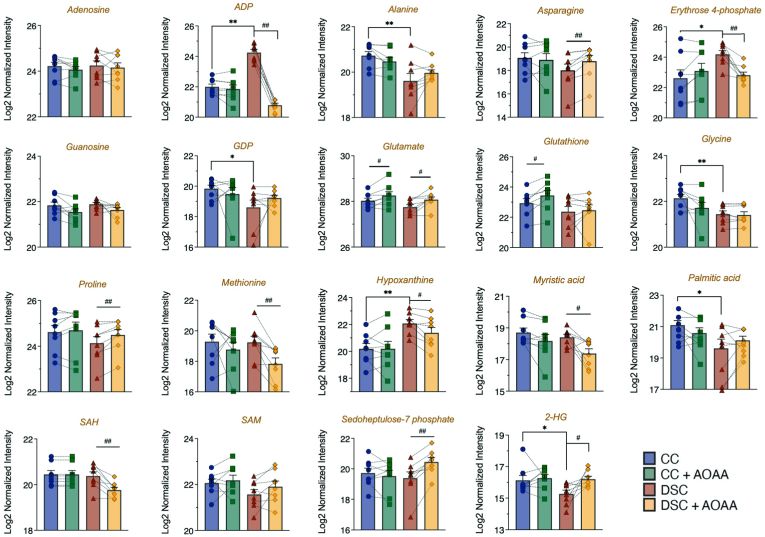
**DS cells exhibit marked dysregulation of metabolism compared to healthy control cells; effect of AOAA.** Various isotopologues (e.g. amino acids, lipids and additional metabolic intermediates) detected by fluxomic analysis are shown in n = 8 human euploid control fibroblasts and n = 8 DS fibroblasts, as summarized in Table 1, with/without AOAA treatment (3 μM). *p ≤ 0.05 and **p ≤ 0.01 indicate significant differences between DSC untreated vs. CC untreated; ^#^p ≤ 0.05 and ^##^p ≤ 0.01 indicate significant differences between CC + AOAA vs. CC untreated or DSC + AOAA vs. DSC untreated.

AOAA in DS cells also increased ^13^C flux into the pentose phosphate pathway intermediate sedoheptulose 7-phosphate (Fig. 8). DS cells exhibited increased ^13^C fluxes into hypoxanthine; an effect, which was normalized by AOAA treatment (Fig. 8). Interestingly, in DS cells, ^13^C flux into the oncometabolite 2-hydroxyglutarate (2-HG) was lower than to control cells; this alteration was also restored by AOAA (Fig. 8). Since 2-HG is produced by isocitrate dehydrogenase (ICDH) and other Krebs cycle enzymes, the inhibition of these enzymes in DS, and the reactivation of the Krebs cycle after CBS inhibition would be consistent with the observed alterations in cellular 2-HG levels in DS. The marked increase in ADP in DS (Fig. 8) was also corrected by AOAA; it is conceivable that DS cells are using up ATP at a faster rate than the rate at which the cells are able to replenish them.

The above results indicate that DS cells exhibit a significant misalignment of biochemical pathways downstream from glucose, and CBS inhibition – with a consequent normalization of cellular H_2_S levels – can prevent correct many of these biochemical alterations. Thus, the current findings would predict that CBS inhibition may provide multiple metabolic and cellular functional benefits in DS. The current fluxomic findings are in partial agreement with recent fluxomic studies recently reported by Anderson and colleagues – which were conducted predominantly in fibroblasts obtained from newborn or young donors [19]. Similar to our results, the Anderson study also reported **(a)** a lower ^13^C flux into alanine in DS than in control cells; **(b)** an increase in various pentose phosphate isobars, which is consistent with our observation of higher Ri/Ru5P ^13^C flux in DS cells than in control cells, confirming a higher flux into the oxidative phase of the PPP in DS than in control (see also above); **(c)** a tendency for lower sedoheptulose 7-phosphate fluxes in DS cells than in control cells, **(d)** a tendency for lower malate levels in DS cells, perhaps indicative of alterations in the aspartate/malate shuttle in DS; **(e)** a tendency for lower a-ketoglutarate levels in DS cells than controls and **(f)** lower alanine levels in DS cells than in controls. However, in contrast to our findings, in the adult DS fibroblast populations, the Anderson study revealed no significant differences between control and DS cells (but a tendency for higher fluxes in DS) for hexose phosphates, and no differences in the ^13^C fluxes into pyruvate were noted between control and DS cells. Importantly, the Anderson study did not directly evaluate carbon fluxes into lactate, and thus it did not directly assess potential shifts between glucose utilization by the Krebs cycle and glycolysis.

Another recent study, utilizing neurospheres derived from embryonic Ts1Cje mouse model of DS (vs. wild-type controls) also reported significant changes in fundamental cellular metabolic pathways and marked alterations in substrate utilization in DS cells [37]. These alterations included an overall suppression of glucose utilization, perturbation in the PPP, lower metabolism of glucose-6-phosphate and various other alterations [37].

**Fig. 9:**
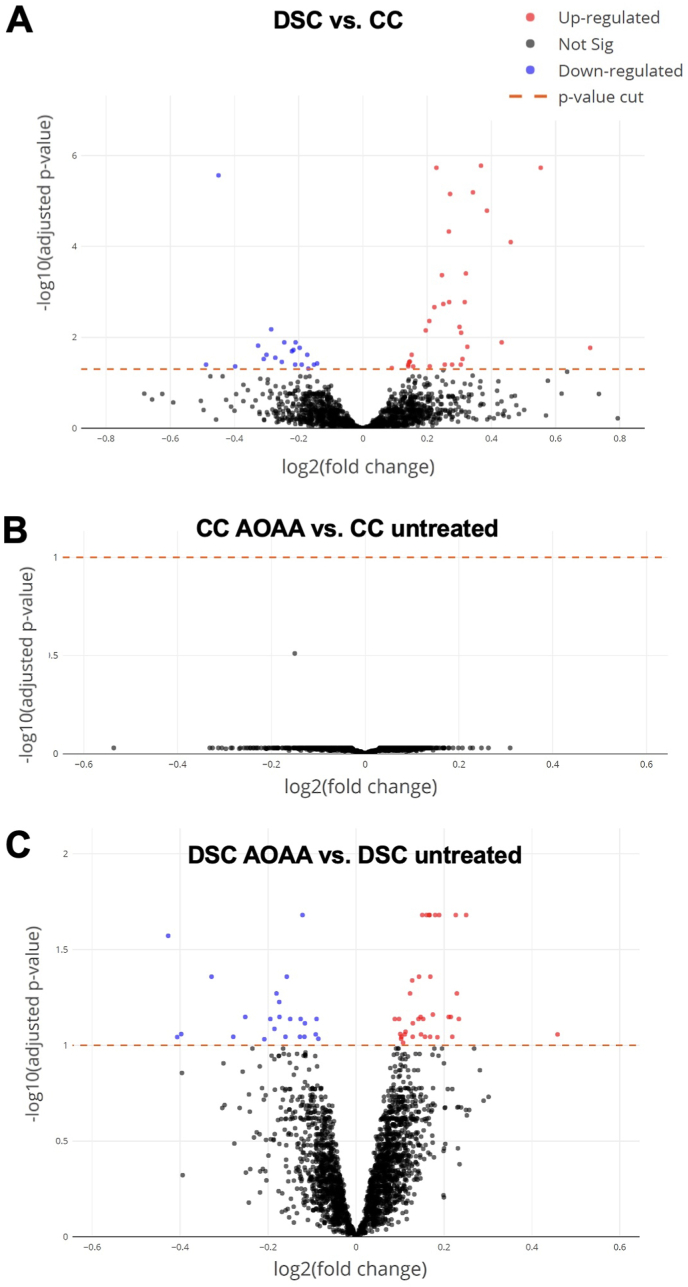
**Volcano plots of proteomic signatures. (A)**: marked differences between DS cells (DSC) and healthy control cells (CC); **(B)**: lack of effect of AOAA in healthy control cells; **(C)**: prominent effect of AOAA in DS cells. "Not Sig" indicates differences that are below the significance level of p<0.05 (black dots). (For interpretation of the references to color in this figure legend, the reader is referred to the Web version of this article.)

**Fig. 10:**
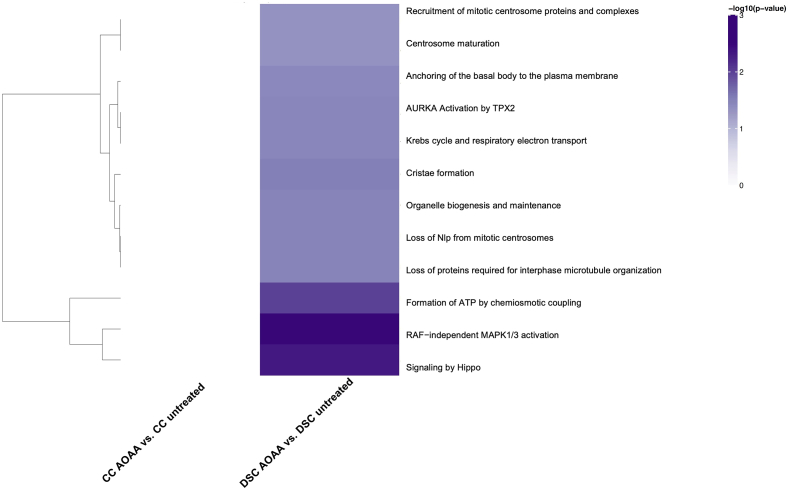
**Heatmap of significantly enriched Reactome Pathways in DS**: Comparisons are shown on the X axis with Reactome Pathways on the Y axis. When performing tests for enrichment, pathways were restricted to include only those with 2 or more genes. Note that for the purposes of display, only Reactome Pathways with an enrichment p-value less than 0.05, were included. Furthermore, only the top 50 pathways are displayed. Color is assigned based on the -log10(enrichment p-value), with lighter colors implying less significant enrichment. Hierarchical clustering was applied to pathways (rows). The most significant pathways were clustered according to Euclidean distance using the complete linkage method. (For interpretation of the references to color in this figure legend, the reader is referred to the Web version of this article.)

**Fig. 11:**
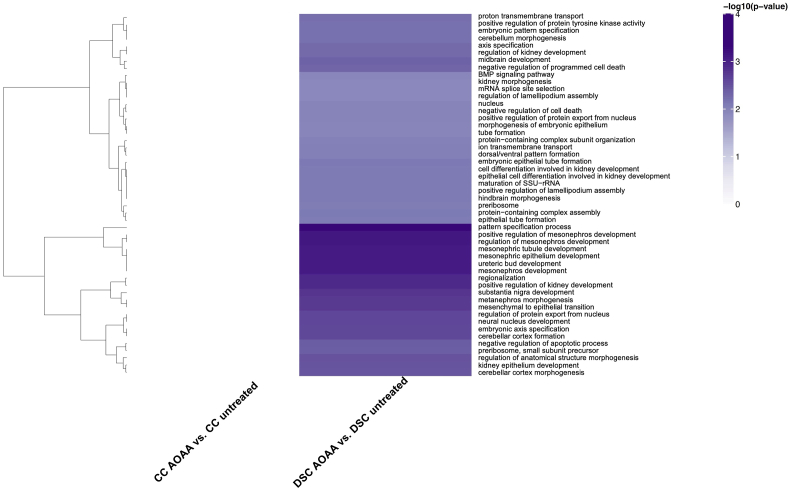
**Heatmap of significantly enriched GO terms in DS:** Comparisons are shown on the X axis with GO terms on the Y axis. When performing tests for enrichment, terms were restricted to include only those with 2 or more genes. Note that for the purposes of display, only GO terms with an enrichment p-value less than 0.05, were included. Furthermore, only the top 50 terms are displayed. Color is assigned based on the -log10(enrichment p-value), with lighter colors implying less significant enrichment. Hierarchical clustering was applied to terms (rows). The most significant terms were clustered according to Euclidean distance using the complete linkage method. (For interpretation of the references to color in this figure legend, the reader is referred to the Web version of this article.)

Control and DS cells (with and without CBS inhibitor treatment) were also subjected to a global untargeted proteomic analysis. This analysis detected many enzymes involved in the interconversion of many of the above analyzed metabolites – as well as a variety of additional proteins, including many enzymes involved in the multitude of metabolic and other cellular processes. The massive differences in the proteome of DS cells vs. control cells are shown in Suppl. Table 1. Similar to prior proteomic analyses of DS cells and tissues [[38], [39], [40]], our analysis demonstrated marked alterations in the proteome of DS cells, affecting, among others, various metabolic pathways, cellular trafficking, DNA structure, stress response, cytoskeleton network, and multiple signaling pathways (Fig. 9, Fig. 10, Fig. 11). In total, 5538 proteins have been reproducibly detected. Approximately 11% of these proteins were significantly different between control and DS group at the significance level of p < 0.05; approximately 56% of these proteins were upregulated and 44% downregulated in DS cells compared to control cells (Fig. 10, Suppl. Table 1). Many of the proteins upregulated in DS were located on Chromosome 21 (according to the expected gene dosage effect). However, a significant number of the affected proteins were located on other chromosomes, in line with prior observations [40,41].

However, the proteomic analysis did not reveal very significant differences in the expression of enzymes involved in the regulation of central carbon metabolism, yielding the overall conclusion is that the changes that we observe in terms of bioenergetics and fluxomics are not principally driven by changes in rate limiting enzyme levels, but, rather, by functional biochemical effects, which may be driven by mechanisms that regulate the catalytic activity, rather than the expression level of these proteins. Among many mediators, H_2_S can induce such effects, for instance through persulfidation of protein cysteines or through its actions on metal-containing active centers [4]. For instance, in cancer cells, H_2_S has been shown to drive SQR-dependent respiration causing a reversal of the TCA cycle, glutamine deprivation, increased glycolysis and lipogenesis – effects that are not ultimately linked to altered expression of SQR protein [42]. In other cell types – e.g. DS cells – excess H_2_S can inhibit mitochondrial Complex IV activity [8] (see also above) without affecting the expression level of Complex IV (or of other electron transport proteins) [8].

Hexokinases are key enzymes which catalyze the conversion of glucose into glucose-6-phosphate. However, hexokinase-1 and hexokinase-2 expression levels were unaffected by DS or AOAA (Table 2). Previous studies [38] have demonstrated increases in hexokinase *activity* in DS; thus, it is likely that this enzyme is activated in DS via some post-transcriptional modification. At the next step of glycolysis, the conversion of glucose-6-phosphate to fructose-6-phosphate is catalyzed by glucose-6-phosphate isomerase. The group of Lubec has previously reported a downregulation of the mRNA for this enzyme in DS brain [43,44]; in our proteomic analysis this enzyme was not differentially expressed between control and DS cells (Table 2). Phosphofructokinase-1 (PFK-1) is one of the most important regulatory enzymes of glycolysis. Since this enzyme is encoded on Chromosome 21, in DS, a gene dosage effect was expected and indeed, a statistically significant (approx. 15–25%) upregulation was detected (Table 2). Prior studies have already recognized the functional role of this alteration in DS [45]. However, PFK-1 expression was not inhibited by AOAA in DS cells (Table 2). Thus, the effect of AOAA on glycolytic carbon fluxes in DS is unlikely to be due to the regulation of the expression levels of this enzyme. Fructose-bisphosphate aldolase (ALDO) is the next enzyme in glycolysis, which metabolizes fructose 1,6-bisphosphate. The fluxomic analysis revealed higher ^13^C fluxes into DHAP in DS cells than in control cells (Fig. 6). However no changes were noted at the expression levels of ALDO (Table 2). Interestingly, ALDO has recently been shown to be sulfhydrated by H_2_S in endothelial cells, possibly representing an activating modification [46]. Thus, a stimulatory effect of H_2_S on this enzyme in DS is possible (but remains to be studied in the future).

**Table 2:** **Expression levels of enzymes involved in the interconversion of isotopologues detected in the fluxomic analysis.** Enzyme levels were determined by proteomic analysis and subjected to Log2 normalization. *p ≤ 0.05 and **p ≤ 0.01 indicate significant differences between DSC untreated vs. CC untreated; ^#^p ≤ 0.05 indicates significant differences between CC + AOAA vs. CC untreated or DSC + AOAA vs. DSC untreated.

Enzyme	Pathway	CC	CC + AOAA	DSC	DSC + AOAA
Hexokinase 1	Glycolysis	13.55 ± 0.03	13.54 ± 0.03	13.53 ± 0.05	13.52 ± 0.05
Hexokinase 2	Glycolysis	11.86 ± 0.05	11.70 ± 0.06	11.76 ± 0.05	11.73 ± 0.06
glucose-6-phosphate isomerase	Glycolysis	13.57 ± 0.18	13.58 ± 0.16	13.70 ± 0.10	13.63 ± 0.12
Phosphofructokinase-1, muscle type	Glycolysis	11.36 ± 0.04	11.36 ± 0.03	11.53 ± 0.03**	11.54 ± 0.02
Phosphofructokinase-1, liver type	Glycolysis	12.46 ± 0.04	12.44 ± 0.03	12.78 ± 0.04**	11.87 ± 0.03^#^
Fructose-bisphosphate aldolase	Glycolysis	10.98 ± 0.09	10.93 ± 0.11	10.83 ± 0.10	10.81 ± 0.09
Glyceraldehyde 3-phosphate dehydrogenase (GAPDH)	Glycolysis	12.59 ± 0.09	12.42 ± 0.04	12.31 ± 0.07*	12.27 ± 0.05
3-phosphoglycerate kinase (3-PGK)	Glycolysis	14.38 ± 0.06	14.38 ± 0.07	14.41 ± 0.07	14.39 ± 0.06
Phosphoglycerate mutase (PGM)	Glycolysis	13.43 ± 0.04	13.42 ± 0.03	13.56 ± 0.03*	13.53 ± 0.03
Enolase 1 (ENO-1)	Glycolysis	15.73 ± 0.06	15.67 ± 0.03	15.69 ± 0.08	15.65 ± 0.04
Enolase 2 (ENO-2)	Glycolysis	11.39 ± 0.06	11.30 ± 0.07	11.37 ± 0.06	11.40 ± 0.05
Pyruvate kinase M1/M2	Glycolysis	15.69 ± 0.04	15.68 ± 0.03	15.74 ± 0.04	15.75 ± 0.04
Lactate dehydrogenase (LDH) A	Glycolysis	14.90 ± 0.04	14.88 ± 0.04	14.96 ± 0.05	14.92 ± 0.05
Lactate dehydrogenase (LDH) B	Glycolysis	13.48 ± 0.04	13.42 ± 0.05	13.38 ± 0.06	13.35 ± 0.03
Pyruvate dehydrogenase E1 subunit alpha	Glycolysis, Krebs cycle	11.40 ± 0.03	11.39 ± 0.03	11.34 ± 0.03	11.33 ± 0.02
Pyruvate dehydrogenase E1 subunit beta	Glycolysis, Krebs cycle	11.82 ± 0.04	11.78 ± 0.02	11.81 ± 0.03	11.80 ± 0.03
Pyruvate dehydrogenase (PDH) complex component X	Krebs cycle	10.39 ± 0.06	10.40 ± 0.05	10.36 ± 0.05	10.35 ± 0.05
Citrate synthase	Krebs cycle	12.60 ± 0.03	12.58 ± 0.03	12.59 ± 0.05	12.57 ± 0.05
Aconitase 1	Krebs cycle	12.47 ± 0.08	12.41 ± 0.07	12.43 ± 0.06	12.45 ± 0.06
Aconitase 2	Krebs cycle	12.51 ± 0.03	12.52 ± 0.02	12.47 ± 0.05	12.45 ± 0.05
Isocitrate dehydrogenase (ICDH) 1	Krebs cycle	13.13 ± 0.06	13.13 ± 0.06	13.33 ± 0.07*	13.27 ± 0.06^#^
Isocitrate dehydrogenase (ICDH) 2	Krebs cycle	12.53 ± 0.09	12.54 ± 0.09	12.64 ± 0.09	12.70 ± 0.08
α-ketoglutarate dehydrogenase (α−KGDH)	Krebs cycle	12.39 ± 0.05	12.36 ± 0.06	12.45 ± 0.04	12.44 ± 0.05
Succinyl-CoA synthetase (S-CoAS) - GDP-forming subunit beta	Krebs cycle	11.02 ± 0.04	10.98 ± 0.03	10.98 ± 0.04	11.01 ± 0.05
Succinyl-CoA synthetase (S-CoAS) - GDP/ADP-forming subunit alpha	Krebs cycle	12.42 ± 0.03	12.42 ± 0.04	12.38 ± 0.04	12.37 ± 0.03
Succinate dehydrogenase (SDH) complex flavoprotein subunit A	Krebs cycle	11.72 ± 0.02	11.69 ± 0.04	11.78 ± 0.05	11.77 ± 0.04
Succinate dehydrogenase (SDH) complex subunit B	Krebs cycle	11.36 ± 0.02	11.30 ± 0.04	11.34 ± 0.03	11.41 ± 0.04
Succinate dehydrogenase (SDH) complex subunit C	Krebs cycle	7.06 ± 0.03	7.13 ± 0.05	7.17 ± 0.05	7.18 ± 0.03
Fumarase	Krebs cycle	12.61 ± 0.03	12.58 ± 0.04	12.59 ± 0.03	12.55 ± 0.02
Malate dehydrogenase 1 (MDH-1)	Krebs cycle	13.03 ± 0.01	13.04 ± 0.03	13.18 ± 0.01**	13.16 ± 0.03
Malate dehydrogenase 2 (MDH-2)	Krebs cycle	13.73 ± 0.05	13.67 ± 0.04	13.79 ± 0.04	13.74 ± 0.05
Glucose-6-phosphate dehydrogenase (G6PDH)	PPP	13.35 ± 0.04	13.31 ± 0.08	13.30 ± 0.04	13.33 ± 0.03
6-phosphogluconate dehydrogenase (6-PGDH)	PPP	13.47 ± 0.05	13.45 ± 0.05	13.47 ± 0.09	13.42 ± 0.08
Glycerol-3-phosphate dehydrogenase (G3PDH)	PPP	12.59 ± 0.09	12.42 ± 0.04	12.31 ± 0.07	12.27 ± 0.05
Glucolactonase	PPP	12.17 ± 0.05	12.17 ± 0.04	12.31 ± 0.03*	12.32 ± 0.03
Transketolase	PPP	13.97 ± 0.05	13.95 ± 0.05	13.92 ± 0.04	13.87 ± 0.04
Phosphoglycerate dehydrogenase	Serine synthesis	13.17 ± 0.08	13.19 ± 0.08	13.18 ± 0.09	13.17 ± 0.09
Phosphoserine phosphatase (PDPH)	Serine synthesis	10.11 ± 0.07	10.10 ± 0.09	10.16 ± 0.13	10.06 ± 0.10
ATP citrate lyase (ACLY)	Fatty acid synthesis	14.33 ± 0.04	14.33 ± 0.04	14.49 ± 0.04	14.49 ± 0.03
Acetyl-CoA carboxylase (ACC)	Fatty acid synthesis	11.46 ± 0.03	11.47 ± 0.03	11.49 ± 0.04	11.51 ± 0.04
Fatty acid synthase (FAS)	Fatty acid synthesis	14.27 ± 0.05	14.28 ± 0.05	14.27 ± 0.04	14.29 ± 0.04
Phosphoribosyl pyrophosphate synthetase (PRPS) 1	NAD^+^ synthesis	12.55 ± 0.12	12.50 ± 0.12	12.60 ± 0.09	12.55 ± 0.11
Phosphoribosyl pyrophosphate synthetase (PRPS) 2	NAD^+^ synthesis	9.96 ± 0.07	9.94 ± 0.06	9.87 ± 0.07	9.82 ± 0.07

In the 6th step of glycolysis, glyceraldehyde 3-phosphate dehydrogenase (GAPDH) catalyzes the conversion of G3P to d-glycerate 1,3-bisphosphate, which, is converted to 1,3-bisphospho-d-glycerate and, further, by 3-phosphoglycerate kinase (PGK) to 3-phosphoglycerate and subsequently to 2-phosphoglycerate by phosphoglycerate mutase (PGM). In our fluxomic analysis, ^13^C fluxes into 3-phosphoglycerate were found to be stimulated in DS and this effect tended to be attenuated by AOAA (Fig. 7). In our proteomic analysis, GAPDH expression was found to be 18% lower and PGM expression was 9% higher in DS than control cells but these changes were not affected by AOAA (Table 2). Importantly, the activity of GAPDH can be readily affected post-transcriptionally, i.e. without any change in absolute protein levels. In fact, there are several studies implicating the regulation of GAPDH via H_2_S-mediated sulfhydration [47,48]. Thus, the DS-associated stimulation of glycolysis (at least in part) may involve H_2_S-induced GAPDH sulfhydration, but this possibility remains to be directly investigated in the future.

Enolase (ENO, also known as phosphopyruvate hydratase) is a metalloenzyme responsible for the catalysis of the conversion of 2-phosphoglycerate (2 PG) to phosphoenolpyruvate (PEP) in the ninth and penultimate step of glycolysis. In the final step, in turn, PEP is catabolized into pyruvate by pyruvate kinase (PK, isoforms M1 and M2). The latter enzyme is subject to multiple levels of regulation, including potential upregulation or activation by H_2_S [49]. In the current experimental system, however, neither DS nor AOAA affected the *protein level* of enolase and pyruvate kinase (PK) M1/M2 (Table 2), which – similar to the case of many other enzymes discussed in earlier paragraphs – does not necessarily mean that the *catalytic activity* of these enzymes remained unaffected.

The conversion of pyruvate to lactate is catalyzed by lactate dehydrogenase (LDH). In our experiments, ^13^C flux into lactate was significantly higher in DS cells than in control cells, and this increase was reversed by treatment of the DS cells with AOAA. However, the expression level of LDH, as determined by proteomics, was not different between control and DS and was not affected by AOAA (Table 2). Nevertheless, it is worth mentioning that LDH is, in fact, subject to H_2_S-mediated sulfhydration and consequent activation on the post-transcriptional level [50], and this action may theoretically also be involved in the DS-associated changes in ^13^C fluxes into lactate.

Although the fluxomics analysis showed major changes in Krebs cycle metabolites, none of the associated enzymes showed different expression levels between control and DS (Table 2). The only exception was isocitrate dehydrogenase 1 (ICDH1), which exhibited a slight upregulation in DS, which was partially attenuated by CBS inhibition; and malate dehydrogenase 1 (MDH-1), which was slightly elevated in DS cells compared to controls, but this change was unaffected by CBS inhibition. It should be pointed out, however, that changes in the protein level of Krebs cycle enzymes (or enzymes of the mitochondrial electron transport chain, or changes in the expression level of various ATP synthase complex components) can not translate into functional differences, *if the mitochondrial electron transport chain is blocked due to the inhibitory effect of H*_*2*_*S on mitochondrial Complex IV*. In this case, the utilization of electron donors by the mitochondria is blocked. Thus, the metabolic processes that serve to produce these donors are functionally suppressed (“backed up”) regardless of the expression level of the proteins involved in the interconversion of the various Krebs cycle metabolites. Nevertheless, it is possible that compensatory changes in the expression of various Krebs cycle or mitochondrial enzymes that would happen in the above scenario, in turn, result in altered metabolic shifts *after the reactivation of the electron transport chain* – i.e. when pharmacological inhibition of CBS “lifts” the inhibition of mitochondrial Complex IV by H_2_S.

From the DS-associated alterations in pyrimidine and purine synthesis, one of the most interesting observations of the current results was the change in ^13^C flux into *de novo* NAD^+^, biosynthesis, because this flux was suppressed by DS and restored by CBS inhibition (Fig. 6). Mammalian NAD ^+^ synthesis occurs through *de novo* and salvage pathways. In mammalian cells, 90% of free tryptophan is metabolized through the kynurenine pathway, leading to the *de novo* synthesis of NAD^+^ [51]. The three different salvage pathways start either from nicotinamide, nicotinic acid, or nicotinamide riboside, with nicotinamide being the major source for the biosynthesis of NAD^+^ in most mammalian cells [51]. Most of these pathways are not “visible” from our current fluxomic analysis. Nevertheless, the pathway by which NAD^+^ is synthesized from ribulose 5-phosphate is detectable. This pathway involves multiple steps, including the action of the ATP-consuming enzyme phosphoribosyl pyrophosphate synthetase (PRPS) as a proximal step, as well as a further step that involves the incorporation of nicotinamide via multiple enzymatic steps [51]. Our current fluxomic analysis did not measure PRPP, but prior studies in DS erythrocytes have demonstrated a slight decline in the cellular concentration of this metabolite [52]. On the protein level, neither PRPS1 or PRPS2 levels were affected by DS. Nevertheless – since the activity of these enzymes is ATP dependent – it is conceivable that an overall improvement of cellular bioenergetic status (see Fig. 1, Fig. 2, Fig. 3) after CBS inhibition may also produce an enhancement of PRPP levels and a consequent stimulation of NAD^+^ biosynthesis.

**Fig. 12:**
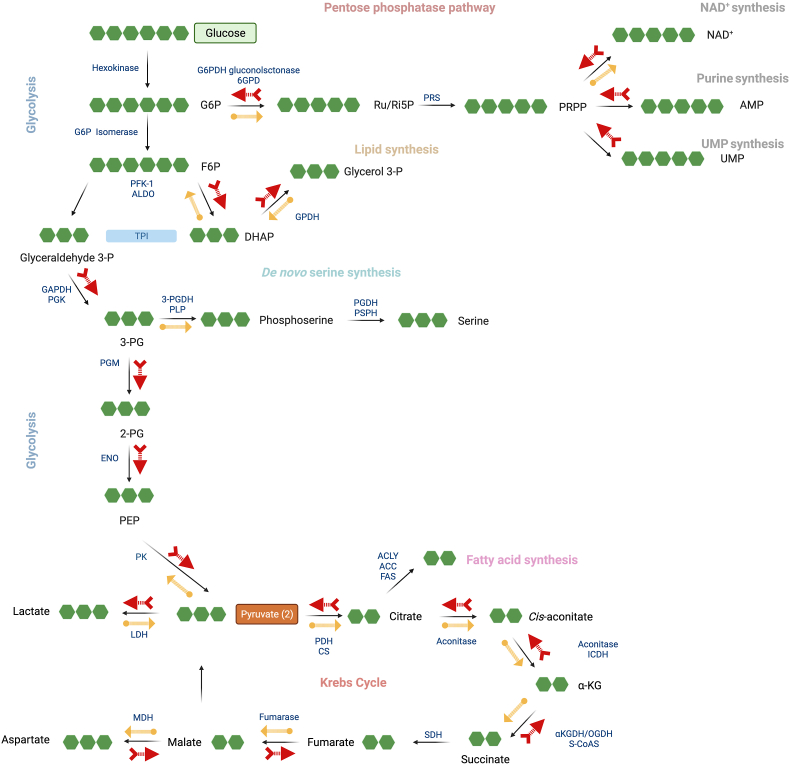
**Summary of the DS-associated central carbon metabolism and related pathways assessed by the fluxomics method.** Blue arrows: normal direction of the fluxes under physiological conditions. Red arrows: shifts induced in DS; yellow arrows: shifts in DS cells induced by AOAA. (For interpretation of the references to color in this figure legend, the reader is referred to the Web version of this article.)

Taken together, the experiments presented and discussed above have identified significant shifts in central carbon metabolism in DS, and demonstrated that the CBS inhibitor AOAA can revert some of these alterations. However, the fluxomic alterations, for the most part, are not driven through changes in the expression of various central carbon metabolism enzymes – although post-transcriptional regulation of some of the enzymes involved in these processes, via H_2_S-mediated sulfhydration remains a possibility. The DS-associated fluxomic shifts central carbon metabolism, and the effects of AOAA on some of these alterations are summarized in Fig. 12.

Some additional proteomic changes that are worth mentioning from a bioenergetic/metabolic/mitochondrial standpoint, are the DS-associated upregulation of phosphofructokinase (PFK) and malate dehydrogenase (MDH) isoforms and of ACLY (an enzyme that is regulated by H_2_S [53]). In addition, carbonyl reductases 1 and 3, phosphoribosyl glycinamide formyltransferase, microsomal glutathione S-transferase 1, pyridoxal kinase, holocarboxylase synthetase, GDP-l-fucose synthase, aldo-keto reductase family 1 member A1, glutamate-cysteine ligase modifier subunit were upregulated in DS; these alterations predicts significant changes in a variety of metabolic processes in DS cells. The DS-associated dysregulation of several mitochondrial ATP synthase components (ATPase H^+^ transporting V1 subunits A and F, ATP synthase F1 subunits gamma and epsilon and ATP synthase peripheral stalk subunit d) is in line with prior observations in various human and animal models of DS demonstrating the dysregulation of multiple ATP synthase complex protein components [[54], [55], [56], [57], [58], [59]]. These changes may represent futile or ineffective compensatory mechanisms if Complex IV is inhibited by H_2_S in DS cells and the entire mitochondrial electron transport is blocked at the distal point of the chain.

We have also noted a significant DS-associated upregulation of Nudix hydrolase 1. This has also implicated in DS before [60] and may predict possible alterations in DNA repair processes in DS. Our observation that DS is associated with an upregulation of protein *O*-fucosyltransferase 2 is in line with the widespread changes in protein fucosylation observed [61] in DS. The upregulation of beta-secretase 2 – in concert with the upregulation of amyloid precursor protein – may be involved in the pathogenesis of DS; they may also be important pathophysiological events linking DS to the pathogenesis of DS-associated Alzheimer's disease [62,63].

We should also briefly highlight those proteins and pathways that the proteomics analysis revealed as ones that *selectively respond to CBS inhibition in DS* (Fig. 9). In the DS cells, AOAA produced a significant enrichment of several classes of proteins, belonging (in decreasing order of intensity) to a number of key cellular pathways, such as Hippo signaling, RAF−independent MAPK1/3 activation, formation of ATP by chemiosmotic coupling, proteins regulating centrosome and microtubule organization and maturation, mitochondrial and other organelle biogenesis and maintenance, Krebs cycle and mitochondrial electron transport chain (Fig. 9, Fig. 10, Fig. 11 and Suppl. Table 1.) The functional consequence of these effects will be a subject of follow-up studies.

**Fig. 13:**
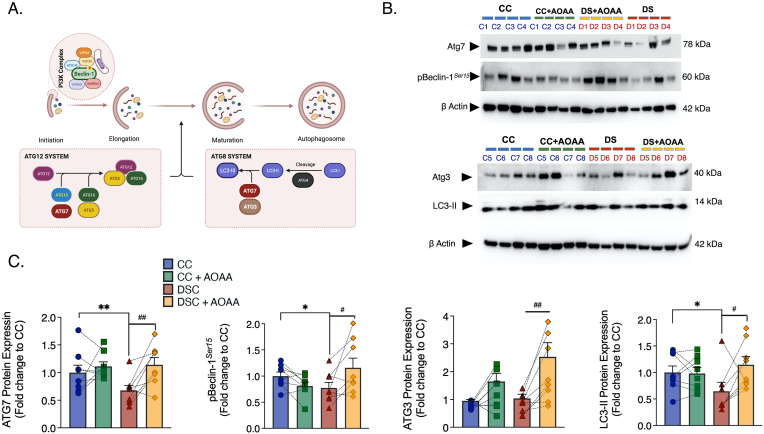
**Pharmacological inhibition of CBS with AOAA attenuates the DS-associated down-regulation of autophagy-related (Atg) components for the formation and maturation of the autophagosome. (A)**: schematic summary of the pathways involved; **(B)**: representative Western blots; **(C)**: Quantification of the protein expression of ATG7, phosphorylated beclin-1 (pBeclin-1) at Ser15, ATG3, and the lipidated, autophagosome-associated LC3-II. β-Actin served as loading control for densitometry. Each bar graph represents the mean ± SEM of n = 8 human euploid control fibroblasts and n = 8 DS fibroblasts. C1–C8 and D1-D8 corresponds to the specific donors listed in Table 1. Dotted connecting lines in the bar graphs indicate the same cell from a specific donor with/without AOAA treatment. *p ≤ 0.05, **p ≤ 0.01 DSC indicates significant differences between DSC untreated vs. CC untreated; ^#^p ≤ 0.05, ^##^p ≤ 0.01 indicates significant differences between DSC + AOAA vs. DSC untreated.

Our analysis, described in the previous sections, as well as multiple independent reports have demonstrated that DS is associated with significant mitochondrial and bioenergetic misalignments. In the current study, CBS inhibition was found to restore mitochondrial function and realign some of the DS-associated alterations in central carbon metabolism. Mitochondrial health relies on a complex net of quality control mechanisms, among which the recycling of damaged or ROS-producing mitochondria via the macroautophagic (or autophagic) route is an important one. This process entails a cytosolic pathway of dynamic membrane rearrangement for the formation of the autophagosome that aims to catabolize long-lived proteins and organelles by fused with the lysosomes and thus to recycle amino acids, lipids, and nucleosides crucial for cellular homeostasis. It is mediated by conserved autophagy-related (Atg) proteins that associate into complexes – the class III phosphatidylinositol 3 kinase (PI3K) complex, the ATG9-membrane complex, and two conjugation systems consisting of the ATG3-ATG8/LC3 and the ATG5-ATG12:ATG16L complex, regulating the biogenesis, elongation, and maturation of the autophagosome, as illustrated in Fig. 13. The abundance and activity of the Atg machinery build upon the two types of PTMs, lysine acetylation and cysteine oxidation, which both depend on intracellular redox status and NAD^+^ availability (reviewed in Ref. [64]). Consistently, we show that the NAD^+^ depletion along with the aberrant ROS generation coincides with a suppressed expression of the Atg components, ATG7 and LC3 of the Atg12 and Atg8 systems and with a down-regulation of the phosphorylation of Beclin-1 for autophagic flux induction in DS fibroblasts (Fig. 13). Diminished autophagic clearance along with lysosomal impairments have been already reported in DS [[65], [66], [67], [68]]. However, the novel aspect of the current study is that CBS inhibition normalizes some of these alterations, thereby further promoting the availability of the ATG3 component, possibly to facilitate the formation of functional complexes of the autophagic machinery (Fig. 13). It is conceivable that some of the above beneficial affects of CBS inhibition on various autophagic parameters may be related to the overall improvement in central carbon metabolism and cellular energy charge.

The current study has several limitations, stemming from a combination of factors. **(1)** First of all, the study investigates a very complex pathophysiological condition. **(2)** Second, we attempted to determine the role of another very complex system, the CBS/H_2_S pathway. **(3)** Third, we have used a selection of control and DS samples, each from a different human subject (different sex, age). **(4)** Fourth, the CBS inhibitor used in the current study has some selectivity/specificity issues.

(1) With respect to the complexity of DS, the topic has already been highlighted in many of the prior sections. DS represents with a combination of “gene dosage effects” (producing an upregulation of genes encoded on Chromosome 21) and secondary, compensatory and/or ‘domino effects’ on other signaling pathways and other proteins and effector pathways (affecting gene products encoded on all other chromosomes). These processes culminate in the dysregulation of thousands of genes and proteins in an age- and cell type-specific fashion. The field has come a long way from the “Lejeune Machine” [69,70], which was an early attempt to integrate some of the pathobiochemical alterations in DS. Nevertheless, it is clear that these analyses are incomplete – for instance, the current project did not include non-target metabolomics analysis, sulfur metabolomics, gene expression analysis, and many other analyses – and remain to be further refined. In addition, the current study only included one type of cell, fibroblast; additional work, for instance primary neurons or induced stem cells, should be conducted to further test the importance of the CBS pathway in DS.
(2) With respect to the complexity of the CBS/H_2_S pathway, we are facing another level of challenge. CBS, as an essential element of the reverse transsulfuration pathway, is intricately interlinked to the metabolism and interconversion of sulfur-containing amino acids, but also regulates many other metabolites including S-adenosylmethionine (SAM), S-adenosylhomocysteine (SAH), glutathione (GSH) and others. It is also interlinked with the 3-MST system, which utilizes the 3-mercaptopyruvate generated by cysteine aminotransferase (CAT) by forming a persulfide on its active site (R–SH to R–SSH). The persulfide, in turn, releases H_2_S in the presence of a reductant (R′-SH), but 3-MST is also a prominent source of polysulfides, which have biological regulatory roles that are biochemically different from those of H_2_S [[71], [72], [73]]. Since not only CBS, but also 3-MST is upregulated in DS, it is clear that DS is associated with a combined overproduction of H_2_S as well as polysulfides, and it is likely that both of these species contribute to the biochemical alterations we have observed.
(3) With respect to complexity of DS, age of the cells studied represents an important additional factor. DS is associated with important developmental misalignments, and it is clear that the pathobiochemistry of DS changes as the individual ages. The age-dependent differences in metabolic processes have recently been highlighted by the recent report by Anderson and co-workers [19]. In the current study, we have studied fibroblasts obtained from 8 healthy controls – which are fairly uniform in terms of their central carbon fluxomic signatures and fibroblasts from 8 DS individuals, mainly newborn or young donors – which are presenting with a larger heterogeneity in their central carbon fluxomic signatures. Combining the cellular responses from these various individuals will, on one hand, increases the “noise” in the system, but on the other hand, improves the biological validity and significance of the experimental findings. In this respect, the DS-associated changes in CBS expression, changes in mitochondrial function and changes in central carbon metabolism appear to be fairly uniform; with the vast majority of samples showing, directionally, the same type of responses (see Fig. 1, Fig. 2, Fig. 3, Fig. 4, Fig. 5, Fig. 6, Fig. 7, Fig. 8). Nevertheless, individualized analysis can also identify outliers; for example, 1 of 8 subjects in DS did not have higher CBS levels in their fibroblasts than the average expression level of all controls; similarly, 3-MST did not appear to be upregulated in 2 of 8 samples (Fig. 1); 2 DS fibroblasts have not exhibited the suppression of mitochondrial O_2_ consumption and the reduction of Complex IV activity that was noted in the rest of the samples (Fig. 3). In contrast, the increase in H_2_S generation was a uniform response in all DS cells (Fig. 1D and E). Thus, all DS cells appear to be under an increased H_2_S “load”, although the relative contribution of the underlying enzymes may be different.
(4) Finally, we should briefly consider the limitations of the pharmacological agent, AOAA. This molecule has been widely used in the literature to inhibit the catalytic activity of CBS in order to study various processes ranging from cancer metabolism to the neurotransmission, vascular function, hypoxia sensing and others, principally due to the fact that available pharmacological options to inhibit CBS are very limited [3,4,34]. However, the effects of AOAA, as far as the modulation of H_2_S homeostasis is concerned, go beyond CBS; this compound, as a broad inhibitor of PLP-dependent enzymes, as well as an inhibitor of another H_2_S-generating enzyme, CSE [74,75]. To our knowledge, the relative effect of AOAA on CSE-vs. CBS-derived H_2_S production has not yet been determined in cell-based models. However, there is no indication that CSE would be upregulated in DS or that CSE would contributes to the pathogenesis of DS. It should also be pointed out that the concentration of AOAA used in our studies (3 μM) is relatively low, compared to a variety of prior *in vitro* studies in various models. At this concentration, inhibition of CBS (which is potently inhibited by AOAA) is expected, but significant inhibition of many other PLP-dependent enzymes (on which AOAA is less potent [75]) is less likely. Importantly, the functional and proteomic results presented in the current study both demonstrated that the pharmacological effects of 3 μM AOAA were more pronounced and, in most cases, directionally different in DS cells than in the control cells. It should be also pointed out that in a subset of the cells studied in the current report (Detroit 551 control cells vs. Detroit 539 DS cells; designated as “C5” and “D5”, respectively), the effect of AOAA (3 μM) has already been compared to siRNA-mediated CBS silencing on mitochondrial O_2_ consumption and Complex IV activity, and the effects were comparable [9]. Importantly, AOAA has also been shown to exert beneficial effects in a rat model of DS *in vivo* [8]. All of the above considerations are consistent with the suggestion that in DS in general, and in the current experimental design in particular, CBS is a principal pharmacological target of AOAA. Nevertheless, the current results should be further extended in future studies using genetic or alternative pharmacological approaches.

## 4 Conclusions

The current report demonstrates fundamental changes in the central carbon metabolism of human DS fibroblasts, that are consistent with the presence of pseudohypoxia, in line with multiple lines of prior observations. There is a blockade of mitochondrial electron transport and aerobic ATP formation in DS cells, which is compensated by a switch to glycolysis. These changes in DS cells are, to a significant part, due to H_2_S overproduction in DS, which is generated by CBS in these cells (which is upregulated due to gene dosage effect). The functional consequences of the H_2_S-driven pseudohypoxia in DS include a reduction in cellular proliferation rate (Fig. 2) and a reduction in the ability of the cells to process proteins, i.e. a dysregulated autophagy response (Fig. 13); these alterations can be reversed by treatment of the cells with AOAA. *In vivo*, the consequence of a CBS/H_2_S-mediated pseudohypoxia in DS can include impairments in neuronal metabolic activity, neurotransmission, neuronal electrical activity generation, and neurobehavioral function and memory generation; according to recent studies, these alterations can also be reversed by genetic correction or pharmacological inhibition of CBS [[7], [8], [9]].

The fluxomic studies presented in the current report indicate that inhibition of H_2_S overproduction in DS cells improves aerobic, mitochondrial ATP generation and *de novo* NAD^+^ biosynthesis; the same intervention also counteracts ROS overproduction and autophagic machinery defects. These effects are expected to fundamentally improve multiple cell functions, which, in turn, would be expected to yield improved cell viability, improved cell differentiation and cellular repair processes, improved processing of proteins (which, in the longer term, may counteract the accumulation of misfolded proteins, a hallmark and key pathophysiological event in various forms of neurodegeneration). All of these findings strengthen the 20-year-old hypothesis (the so-called “Kamoun hypothesis” [5]) that H_2_S overproduction, via a metabolic inhibitory effect plays a pathogenetic role in DS and support the concept of pharmacological inhibition of CBS as a potential future experimental therapeutic approach in DS.

## Ethics approval and consent to participate

The studies are based on publicly available human cell types and did not require additional ethic committee approval.

## Consent for publication

The paper does not contain any individual person's data in any form.

## Availability of data and materials

The data that support the findings of this study are available from the corresponding author, C.S. upon reasonable request.

## Funding

This work was supported by grants from the Jerome LeJeune Foundation (Paris, France). The FIMM Metabolomics Unit was supported by 10.13039/100015735HiLIFE and 10.13039/501100013840Biocenter Finland.

## Authors' contributions

Experimental design: TP, LP, EBR, AIN, CS. Experimentation: TP, LP, EBR, AIN. Data analysis: TP, LP, EBR, AIN. Manuscript writing: TP, LP, EBR, AIN CS.

## Declaration of competing interest

The authors have no conflict of interest to declare.

## List of abbreviations

α−KG: α-ketoglutarate
α−KGDH: α-ketoglutarate dehydrogenase
2-HG: 2-hydroxyglutarate
3-MST: 3-mercaptopyruvate sulfurtransferase
3-MP: 3-mercaptopyruvate
3-PG: 3-phosphoglyceric acid
3-PGK: phosphoglycerate kinase
6-GPDH: 6-phosphogluconate dehydrogenase
6-PGDH: 3 phosphoglycerate dehydrogenase
ACC: acetyl-CoA carboxylase
ACLY: ATP citrate lyase
AD: Alzheimer's disease
ALDO: fructose-biphosphate aldolase
AOAA: aminooxyacetic acid
ATP: adenosine 5′-triphosphate
AzMC: 7-azido-4-methylcoumarin
BrDU: 5-bromo-2′-deoxyuridine
BSA: bovine serum albumin
CAT: cysteine aminotransferase
CBS: cystathionine β−synthase
CC: control cell/euploid fibroblast
CSE: cystathionine γ−lyase
DCF: 2′, 7′-dichlorofluorescein
DCFDA: 2′,7′-dichlorofluorescein diacetate
DHAP: dihydroxyacetone phosphate
DS: Down syndrome
DSC: dermal fibroblasts from individuals with Down syndrome
DYRK1A: dual specificity tyrosine phosphorylation regulated kinase 1A
ENO: enolase
ETHE-1: ethylmalonic encephalopathy 1 protein
F6P: fructose-6-phosphate
FAS: fatty acid synthase
FBS: fetal bovine serum
FCCP: carbonyl cyanide 4-(trifluromethoxy)phenylhydrazone
G2P: glycerol-2-phosphate/2-phosphoglycerate
G3P: glycerol-3-phosphate/3-phosphoglycerate
G3PDH: glycerol 3 phosphate dehydrogenase
G6P: glucose-6-phosphate
G6PDH: glucose-6-phosphate dehydrogenase
GABA: gamma-aminobutyric acid
GABA-T: gamma-aminobutyric acid transaminase
GAPDH: glyceraldehyde phosphate dehydrogenase
GO: gene ontology
GOT: aspartate transaminase
GSH: glutathione
H-ESI: heated electrospray ionization
H_2_S: hydrogen sulfide
HPLC: high-performance liquid chromatography
ICDH: isocitrate dehydrogenase
LDH: lactate dehydrogenase
MDH: malate dehydrogenase
MS: mass spectrometry
NAD: nicotinamide adenine dinucleotide
OCR: oxygen consumption rate
P3: (E)-2-(3-(6-(2-hydroxyethylamino)naphthalen-2-yl)-3-oxoprop-1-enyl)-3,5-dimethoxybenzaldehyde
PBMC: peripheral blood mononuclear cell
PBS: phosphate-buffered saline
PCR: polymerase chain reaction
PDH: pyruvate dehydrogenase
PEP: phosphoenolpyruvate
PER: proton efflux rate
PFK-1: phosphofructokinase-1
PGM: phosphoglycerate mutase
PK: pyruvate kinase
PLP: 3 phosphoserine-aminotransferase
PPP: pentose-phosphate pathway
PRPS: phosphoribosyl pyrophosphate synthetase
PSPH: phosphoserine phosphatase
ROS: reactive oxygen species
Ri5P: ribose 5-phosphate
Ru5P: ribulose 5-phosphate
SAM: S-adenosylmethionine
SAH: S-adenosylhomocysteine
S-CoAS: succinyl-CoA synthetase
SDH: succinate dehydrogenase complex
SEM: standard error of the mean
SQR: sulfide:quinone oxidoreductase
TST: thiosulfate-dithiol sulfurtransferase
UPLC: ultra-performance liquid chromatography
XF: extracellular flux
XTT: 2,3-bis-(2-methoxy-4-nitro-5-sulfophenyl)-2H-tetrazolium-5-carboxanilide

## Appendix A Supplementary data

Suppl. Table 1 Full proteomic data in CC and DSC with and without AOAA treatment. “ischchr 21” indicates proteins encoded on Chromosome 21. Suppl. Table 1
